# Evaluation of levator ani muscle elasticity after vaginal delivery and cesarean section using shear wave elastography

**DOI:** 10.1007/s10396-023-01369-w

**Published:** 2023-09-23

**Authors:** Yoshiyuki Okada, Chie Nakagawa, Miwa Shigeta, Yukiko Nomura, Eisuke Inoue, Kiyotake Ichizuka, Yasukuni Yoshimura

**Affiliations:** 1 Department of Female Pelvic Health Center, Showa University Northern Yokohama Hospital, 35-1 Chigasaki-Chuo, Tsuzuki-Ku, Yokohama City, Kanagawa 224-8503 Japan; 2 Department of Obstetrics and Gynecology, Showa University Northern Yokohama Hospital, Kanagawa, Japan; 3 Showa University Research Administration Center, Tokyo, Japan

**Keywords:** Shear wave elastography, Levator ani muscle, Postpartum, Elasticity

## Abstract

**Purpose:**

The risk of pelvic floor muscle injury is commonly considered to be higher in vaginal than in cesarean delivery. This study aimed to compare levator ani muscle (LAM) elasticity after vaginal and cesarean delivery using shear wave elastography (SWE).

**Methods:**

Postpartum women who underwent a single SWE evaluation 1 month after their first delivery were divided into vaginal and cesarean delivery groups. The elastic moduli of both sides of the LAM were measured in a horizontal section and compared between the groups. In addition, a subgroup analysis was performed to compare LAM elasticity according to the delivery method within the vaginal delivery group—normal vaginal delivery, episiotomy, and operative vaginal delivery.

**Results:**

Sixty-two women were included (vaginal delivery, n = 47; elective cesarean section, n = 15). Multiple regression analysis revealed that the LAM elastic modulus was significantly lower in the vaginal delivery group than in the cesarean delivery group (right LAM: 44.2 vs. 72.7 kPa, p = 0.0036; left LAM 40.4 vs. 82.7 kPa, p < 0.0001). In the subgroup analysis, the right LAM elastic modulus was significantly lower in the operative vaginal delivery subgroup than in the normal vaginal delivery subgroup (p = 0.0131). However, there was no significant difference in the left LAM elastic modulus between the three subgroups.

**Conclusion:**

LAM elasticity was significantly lower after vaginal delivery than after cesarean delivery. Furthermore, the elasticity of the right LAM was lower after operative vaginal delivery than after normal vaginal delivery. SWE has the potential to provide an objective quantitative assessment of postpartum pelvic floor muscle recovery.

## Introduction

Vaginal delivery is usually associated with a higher risk of pelvic floor muscle damage than cesarean section [1, 2]. This difference is thought to be caused by the avulsion or relaxation of the levator ani muscles (LAMs) [3]. In clinical practice, when examining postpartum women, particularly when palpating the LAMs after vaginal delivery, these muscles are subjectively and empirically found to be relaxed; however, there is no objective indicator of this relaxation.

In addition, episiotomy and operative interventions, such as vacuum or forceps delivery, are reported to be risk factors for damage to the LAMs [4, 5]. Although morphological evaluation of the postpartum LAMs is performed, objective quantitative evaluation, such as postpartum elasticity assessment, is rarely performed.

Shear wave elastography (SWE) enables measurement of tissue elasticity based on the propagation velocity of shear waves generated inside tissues by acoustic radiation pressure [6].

In this study, we hypothesized that LAM elasticity is lower after vaginal delivery than after cesarean section and examined this hypothesis using SWE. We also used SWE to examine the differences in LAM elasticity between normal vaginal delivery, vaginal delivery with episiotomy, and operative vaginal delivery with episiotomy.

## Materials and methods

This was a cross-sectional observational study that included a total of 62 women who visited our hospital 1 month after their first delivery between October 2022 and February 2023. LAM elasticity was compared among 47 women who underwent vaginal delivery and 15 women who underwent cesarean section. Adjustments were made for age, week of delivery, and neonatal weight, which are considered to influence the choice of delivery method and LAM elasticity.

The effects of episiotomy and operative vaginal delivery on LAM elasticity were examined in the 47 participants in the vaginal delivery group, who were divided into three subgroups: normal vaginal delivery (NVD, n = 11), episiotomy (n = 27, all with right mediolateral incision), and operative vaginal delivery (n = 9; vacuum [n = 2] and forceps [n = 7] delivery, all with right mediolateral incision). This was adjusted for the week of delivery and neonatal weight, which can potentially affect LAM elasticity in vaginal delivery.

To reduce potential confounding effects, we excluded participants who delivered earlier than 34 weeks, were parturient, and required conversion to emergency cesarean section because of fetal distress or obstructed delivery after onset of labor.

**Fig. 1:**
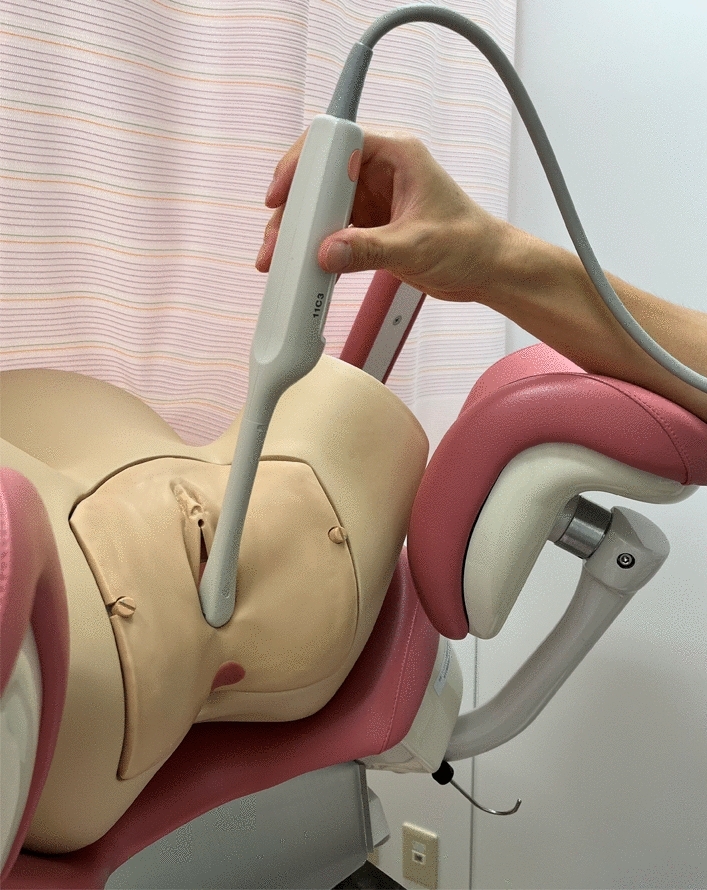
A transvaginal ultrasound probe is placed on the posterior vaginal wall

**Fig. 2:**
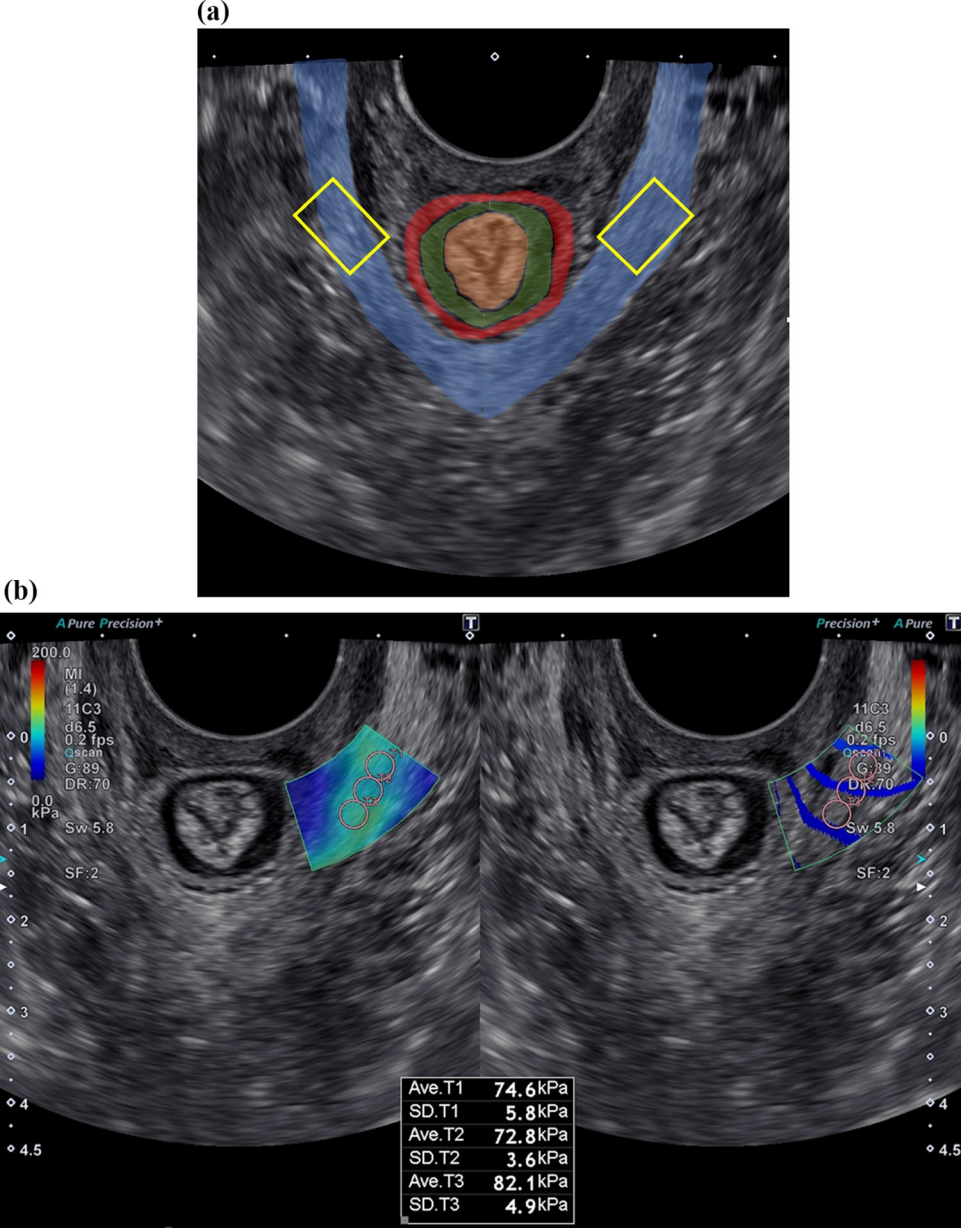
B-mode ultrasound images showing the elasticity measurement procedure. **a** The internal and external anal sphincter and the levator ani muscle are visualized in the axial plane. From the inside, the orange area corresponds to the anal mucosa, the green portion indicates the internal anal sphincter, the red area indicates the external anal sphincter, and the blue the levator ani muscle. The region of interest for shear wave elastography measurement (yellow) is placed on the levator ani muscle just outside the external anal sphincter (at the 3 and 9 o'clock levels) to measure elasticity. **b** Measurement of elasticity using shear wave elastography. While confirming that the shear wave propagates uniformly on the propagation map (right), the elasticity data of the target area are calculated by setting the measurement region of interest (5 mm) at three consecutive points on the color map (left)

LAM elasticity was measured in the lithotomy position with the bladder empty. The ultrasound probe was placed on the posterior vaginal wall (Fig. 1), and an image was obtained using B-mode ultrasound, in which the internal and external anal sphincter and LAM were visualized (Fig. 2a). The SWE acquisition sample frame (region of interest) was placed on the LAM just outside the external anal sphincter (at the 3 and 9 o'clock levels), and a push pulse was applied. To confirm that the shear wave propagated uniformly in the propagation map of the obtained image, the elasticity data of the target area were calculated by setting the measurement region of interest (5 mm) at three consecutive points on the color map, and the average value was determined (Fig. 2b) [7]. In this procedure, the beams emitted from the probe and muscle fibers were oriented perpendicularly to each other as much as possible. Participants were instructed not to exert any force on the LAM.

In a previous study [8], LAM elasticity was measured via the transperineal approach using a convex probe; however, since the elasticity of the LAM may differ depending on the measurement point, we decided to use the external anal sphincter as a marker for measurement to unify the measurement points as much as possible. The ultrasound system used was an Aplio i700 (Canon Medical, Japan). A transvaginal probe (PVT-781VTE) with a center frequency of 7 MHz was used. The elasticity range of SWE was 0–200 kPa. All measurements were performed by a single urogynecologist with more than 1 year of SWE measurement experience. The urogynecologist was blinded to the delivery method; however, complete blinding was not possible because the participants’ group allocation could be assumed based on the condition of their perineum. For reliability analysis, 20 women were assessed in both of their LAMs twice 1 month after delivery.

Participant characteristics were compared between the groups using the Wilcoxon rank-sum test for continuous variables and the chi-square test for categorical variables. Comparisons between groups with respect to the elastic modulus were performed using standard multivariable linear regression analysis. JMP Pro (ver. 16; SAS Institute, Cary, NC, USA) was used for statistical analyses. Statistical significance was set at p < 0.05. For the analysis of intra-rater reliability for ultrasound measurements, we used the intraclass correlation coefficient (ICC) in the R software program (ver. 4.2; Saitama Medical Center, Jichi Medical University, Saitama, Japan) [9].

All procedures performed in the present study were carried out in accordance with the ethical standards of the responsible committees on human experimentation (institutional and national) and the Helsinki Declaration, as revised in 2013. The study protocol was reviewed and approved by the Ethics Committee of Showa University (approval number: 22-037-A), and informed consent was obtained from all participants.

## Results

The characteristics of the participants are presented in Table 1. Age (years; median [interquartile range, IQR]) was significantly lower in the vaginal delivery group than in the cesarean delivery group (33 [29–35] vs. 39 [33–43], p = 0.0002), whereas other parameters were not significantly different between the two groups.

**Table 1:** Characteristics of the study population

	Vaginal delivery (n = 47)	Cesarean section (n = 15)	p-value^*^
Age, years [median (IQR)]	33 (29–35)	39 (33–43)	0.0002
Height, cm [median (IQR)]	158 (154–162)	158 (155–162)	0.74
Non-pregnant body weight, kg [median (IQR)]	50.8 (47.0–56.5)	54.0 (49.0–56.0)	0.12
Non-pregnant BMI, kg/m^2^ [median (IQR)]	19.9 (18.7–22.9)	21.2 (20.4–22.9)	0.06
Body weight 1 month after delivery, kg [median (IQR)]	55.2 (49.4–61.4)	55.6 (50.5–62.5)	0.45
BMI 1 month after delivery, kg/m^2^ [median (IQR)]	21.7 (20.0–24.7)	21.4 (20.9–24.3)	0.78

*IQR* interquartile range, *BMI* body mass index

^*^Wilcoxon rank-sum test

The perinatal data are shown in Table 2. The time of delivery (weeks; median [IQR]) was significantly later in the vaginal delivery group than in the cesarean delivery group (39 [39−40] vs. 37 [36–38], p < 0.0001) since most cesarean sections were planned before the expected date of delivery. Accordingly, neonatal weight (g; median [IQR]) was greater in the vaginal delivery group than in the cesarean delivery group (3000 [2796–3280] vs. 2722 [2534−3018], p = 0.0415). No other differences were observed in terms of neonatal sex or head length.

**Table 2:** Perinatal data

	Vaginal delivery (n = 47)	Cesarean section (n = 15)	p-value
Week of delivery, weeks [median (IQR)]	39 (39–40)	37 (36–38)	< 0.0001*
Sex, female [n (%)]	17 (36.2%)	4 (26.7%)	0.49 ^†^
Neonatal body weight, g [median (IQR)]	3000 (2796–3280)	2722 (2534–3018)	0.0415*
Neonatal head length, cm [median (IQR)]	33.5 (32.5–35.0)	34.0 (33.0–34.5)	0.64*

*IQR* interquartile range

*Wilcoxon rank-sum test

^†^chi-square test

The results of LAM elastic modulus measurements are listed in Table 3. After adjusting for age, week of delivery, and neonatal weight, standard multivariable linear regression analysis revealed that the elastic modulus [kPa (least squares mean)] of the LAM was significantly lower in the vaginal delivery group than in the cesarean section group (right LAM 44.2 vs. 72.7, between-group difference [95% confidence interval, CI] 28.5 [9.7–47.3], p = 0.0036; left LAM: 40.4 vs. 82.7, between-group difference [95% CI] 42.3 [24.9–59.6], p < 0.0001).

**Table 3:** Elasticity modulus of the levator ani muscle (after vaginal delivery vs. cesarean section)

	Vaginal delivery (n = 47)	Cesarean section (n = 15)	Between-group difference **(**95% CI**)**	p-value^*^
Right LAM, kPa	44.2 †	72.7 †	28.5 (9.7–47.3)	0.0036
Left LAM, kPa	40.4 †	82.7 †	42.3 (24.9–59.6)	< 0.0001

*LAM* levator ani muscle, *CI* confidence interval

^†^Least squares mean

^*^Standard multivariable linear regression analysis (adjusted for age, weeks of delivery, and newborn body weight)

**Fig. 3:**
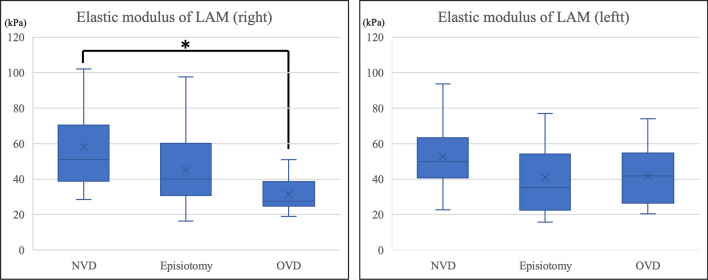
Comparison of LAM elasticity among the vaginal delivery subgroups (NVD vs. episiotomy vs. operative vaginal delivery). Multiple regression analysis is performed, adjusting for weeks of delivery and newborn body weight (p = 0.0131*). *LAM* levator ani muscle, *NVD* normal vaginal delivery, *OVD* operative vaginal delivery

The comparison among the three vaginal delivery subgroups (NVD, episiotomy, and operative vaginal delivery) is shown in Fig. 3 and Table 4. Standard multivariable linear regression analysis adjusted for week of delivery and neonatal weight revealed that the elastic modulus of the right LAM (least squares mean) was 57.3 kPa in the NVD, 44.7 kPa in the episiotomy, and 33.7 kPa in the operative vaginal delivery group, indicating that the elastic modulus of the right LAM was significantly lower in the operative vaginal delivery group than in the NVD group (intergroup difference: 23.6, 95% CI 5.2–41.9, p = 0.0131). The elastic modulus of the left LAM was 52.1 kPa in the NVD, 42.2 kPa in the episiotomy, and 39.4 kPa in the operative vaginal delivery group, with no differences among the groups. There were no cases of fourth-degree perineal tears in this study, and only one case of third-degree perineal tears was observed after forceps delivery.

**Table 4:** Comparison of LAM elasticity among the vaginal delivery subgroups (normal vaginal delivery vs. episiotomy vs. operative vaginal delivery)

	NVD (n = 11)	NVD vs. episiotomy			NVD vs. OVD		
		Episiotomy (n = 27)	Group difference (95% CI)	p-value^*^	OVD (n = 9)	Group difference (95% CI)	p-value*
Right LAM, kPa	57.3 †	44.7 †	12.5 (− 2.4 to 27.5)	0.10	33.7†	23.6 (5.2–41.9)	0.0131
Left LAM, kPa	52.1 †	42.2 †	9.9 (− 3.7 to 23.5)	0.15	39.4†	12.6 (− 4.1 to 29.3)	0.13

*LAM* levator ani muscle, *NVD* normal vaginal delivery, *OVD* operative vaginal delivery, *CI* confidence interval

^†^least square mean

^*^Standard multivariable linear regression analysis (adjusted for weeks of delivery and newborn body weight)

Intra-rater ICC (1,1) values for the LAM elastic modulus measurements were 0.83 (95% CI 0.63–0.93) for the right and 0.88 (95% CI 0.74–0.95) for the left.

## Discussion

To the best of our knowledge, this is the first study to compare the elasticity of the LAM using SWE after different delivery methods.

Vaginal delivery is a major risk factor for pelvic floor disease, including pelvic organ prolapse. Vaginal delivery is considered to have a larger impact on the pelvic floor muscles than cesarean section. The LAM is stretched by two to three times its length during vaginal delivery [3, 10], and further tearing may occur [11]. These changes in the LAM are associated with the future development of pelvic organ prolapse [12, 13]. In addition, an enlarged genital hiatus, as indicated by ballooning, is associated with pelvic floor dysfunction [14]. Thus, while morphological evaluation of the LAM is very important and well-performed, functional evaluations, such as stiffness measurement, have not yet been fully evaluated.

In this study, we considered that evaluating the elasticity of the LAM using SWE could be a new approach to be used in addition to conventional morphological evaluation such as ballooning and avulsion [12, 14]. The evaluation of tissue elasticity using SWE is already widely used in practice and has become a parameter for treatment strategies in the liver [15, 16] and breast areas [17]. It has also been applied in the musculoskeletal (orthopedic) field, as well as in measuring respective mechanical properties of organs and adipose tissue. These previous studies are important for understanding SWE results in the pelvic floor muscles. One study found that the elastic modulus of the Achilles tendon was significantly lower in the Achilles tendinopathy group than in the healthy group, indicating that the elastic modulus was lower when the muscle was damaged [18, 19]. Another study found that the elastic modulus of the gastrocnemius muscle was lower during relaxation than during contraction, indicating that the elastic modulus was lower when the muscle was relaxed [20].

Recent studies that evaluated pelvic floor muscle elasticity using ultrasound SWE reported that the elastic modulus of the LAM increased during the Valsalva maneuver, that is, when the LAM was stretched [8, 21]. Hence, the lower elastic modulus of the LAM after vaginal delivery may reflect relaxation rather than stretching of the LAM. Additionally, after vaginal delivery, not only elasticity but also factors such as edema and inflammation are relevant. Morphological evaluations, such as injury to the LAM, were not performed simultaneously in this study; however, we believe that these factors are also related to the lower elasticity of the LAM, similar to their contribution to low elasticity in Achilles tendonitis.

Although an association between operative vaginal delivery and injury to the LAM has been reported in a meta-analysis [5], the results of that study also inferred that operative vaginal delivery with right mediolateral episiotomy affected the right LAM. All episiotomies involved right mediolateral incisions, and all participants who underwent operative vaginal delivery underwent right mediolateral episiotomy at the same time in our study. The comparison among the three subgroups in the vaginal delivery group showed that the elastic modulus of the right LAM was significantly lower in the operative vaginal delivery group than in the NVD group, while no differences were observed in the left LAM elasticity. This difference may be attributed to the pressure exerted by the fetal head, which significantly affects the right LAM. Since a mediolateral episiotomy cuts the perineal muscles (i.e., the bulbospongiosus and superficial transverse perineal muscles) [22], the buffer between the fetal head and the right LAM may be reduced and the pressure of the rapidly descending fetal head may be directly applied on the right LAM during operative vaginal delivery. However, these speculations cannot be verified without comparison with operative vaginal delivery combined with left mediolateral episiotomy. Although an association between episiotomy and injury to the LAM remains controversial [4], the present results also showed no association between episiotomy and LAM elasticity.

The results of this study showed that LAM elasticity was significantly lower after vaginal delivery than after cesarean section, which supported our hypothesis. The fact that the LAM relaxes more after vaginal delivery than after cesarean section is consistent with the results of this SWE study, suggesting that SWE is a useful method for quantitative assessment of LAM status during the postpartum period. Furthermore, the high ICC of 0.83 and 0.88 as an assessment of the reliability of SWE in the LAM is another indication of the usefulness of SWE. We believe that SWE of the pelvic floor muscles may be a potential indicator for interventions, such as physical therapy, for pelvic floor recovery in the postpartum period and early intervention for the prevention of future pelvic floor disease. It may also be useful for improving the effectiveness of physical therapy and other treatments.

The strength of the present study is that, to the best of our knowledge, it is the first to compare the elasticity of the LAM in the early postpartum period using SWE. However, the study also had some limitations. First, we were not able to evaluate LAM damage at the same time. Thus, we could not confirm in detail whether the low elasticity of the LAM after vaginal delivery was due to reduced elasticity of the LAM or damage to the LAM. Both morphological and elasticity evaluations will be performed simultaneously in future studies. Second, since this study used a two-dimensional measurement method, the measurement primarily focused on the outer portion of the puborectalis muscle in the LAM and did not adequately evaluate the overall elasticity including the pubococcygeus and iliococcygeus muscles. Third, the study had a small sample size and was conducted at a single institution. Finally, due to the nature of the procedures, complete blinding was not possible.

## Conclusion

The elasticity of the LAM was significantly lower after vaginal delivery than after cesarean section and could be objectively evaluated using SWE. In addition, the elasticity of the right LAM was lower after operative vaginal delivery than after NVD, suggesting an effect of operative vaginal delivery on the LAM. SWE has the potential to provide an objective quantitative assessment of postpartum pelvic floor muscles. This method may be applied in the evaluation of pelvic floor muscle recovery in the future, and new insights into postpartum pelvic floor muscle recovery may be obtained by observing the elastic modulus of the pelvic floor muscles over time in addition to morphological evaluation.

## Data Availability

The data that support the findings of this study are available from the corresponding author upon reasonable request.
